# Association between oral health-related quality of life and depressive symptoms in Chinese college students: Fitness Improvement Tactics in Youths (FITYou) project

**DOI:** 10.1186/s12955-019-1163-4

**Published:** 2019-06-04

**Authors:** Zheng Zhang, Ying Tian, Fei Zhong, Cai-fu Li, Shu-mei Dong, Yan Huang, Xing-er Liu, Cong Huang

**Affiliations:** 1 Department of Rehabilitation Engineering Technology, College of Health and Agiculture, Hangzhou Wanxiang Polytechnic, 896 Xixi Road, Hangzhou, 310023 China; 2 College of Sports Science, Shenyang Normal University, 253 Huanghe North Street, Shenyang, 110034 China; 3 Department of Sports and Exercise Science, College of Education, Zhejiang University, 148 Tianmushan Road, Hangzhou, 310007 China; 4 University Hospital of Shenyang Normal University, 253 Huanghe North Street, Shenyang, 110034 China; 5 Division of Physical Education, Hangzhou Shidai Primary School Tianducheng Campus, 9 Tianxing Street, Hangzhou, 311100 China; 6 Kunshan Care Hearts Social Work Center, 1000 Qianjin East Road, Kunshan, 215300 China; 7 Department of Medicine and Science in Sports and Exercise, Tohoku University Graduate School of Medicine, 2-1 Seiryo- machi, Aoba-ku, Sendai, 980-8575 Japan

**Keywords:** Depressive symptoms, Oral health-related quality of life, Youth, Epidemiology

## Abstract

**Background:**

This study aimed to investigate a gender-specific association between oral health-related quality of life (OHRQoL) and depressive symptoms in college students, as there are limited relevant studies conducted among youths.

**Methods:**

In 2017, a cross-sectional study of 3461 Chinese college students was conducted in Shenyang, China. OHRQoL and depressive symptoms were screened by a 14-item oral health impact profile questionnaire and a Self-rating Depression Scale, respectively. A multivariable logistic regression analysis was performed to examine the association of OHRQoL with depressive symptoms.

**Results:**

The number of youths reported to have depressive symptoms was 20.7%. A univariate analysis showed that categories with a OHRQoL score over 6 were more likely to have a higher prevalence of depressive symptoms compared to the category with a score of 0 (male: ORs [95% CI]: 3.10, 2.05–4.68, *P* < 0.001; female: ORs [95% CI]: 3.11, 2.38–4.05, *P* < 0.001). Similar results were observed after adjusting for sociodemographic, anthropometric, and lifestyle-related covariates (male: ORs [95% CI]: 3.07, 1.98–4.76, *P* < 0.001; female: ORs [95% CI]: 2.90, 2.21–3.81, *P* < 0.001).

**Conclusions:**

College students who have higher OHRQoL tend to have a lower prevalence of depressive symptoms.

## Introduction

Depression is a common mental disorder, characterized by feelings of sadness, guilt, or low interest and self-worth, feelings of tiredness, and poor concentration, poor appetite, or disturbed sleep. It has been reported that the incidence of depression was 4.4%, and the population suffering from depression reached 300 million globally [1]. In China, the prevalence of depression was around 4.2%, and the number of cases ranked second worldwide with over 54 million people by 2015. Depressive symptoms are the most important contributor to suicidal behavior [2, 3], which is one of the leading causes of death [4], especially among the adolescent and younger adults population [5]. Furthermore, depression is a type of hard-to-cure disease, since evidence has shown that the relapse rate of major depression was 5% within 6 months, 12% within 1 year, and 33% within 4 years among adolescents who had recovered [6]. Therefore, it is necessary to raise awareness and concerns to devise prevention strategies of depressive symptoms.

As a public health concern, oral disease in adolescents is also a serious burden globally as approximately 60–90% of schoolchildren are affected by dental caries [7], which could have a negative impact on physical, mental, and social functioning [8]. In fact, oral health-related quality of life (OHRQoL) has been verified as relevant to depressive symptoms in school youths, despite only a few studies which have addressed this issue [9–11]. A cross-sectional study found that 145 Brazilian students aged 8–14 years with depressive symptoms were more likely to report poorer OHRQoL [10]. Furthermore, depressive symptoms were inversely associated with OHRQoL among 1200 American youths aged 7–18 years with cleft palate [11]. However, similar studies have not been conducted on Asian youths. Meanwhile, it is unclear whether depressive symptoms are related to OHRQoL in youths screened by the 14-item Oral health impact profile (OHIP-14) [12], which is a scale measuring perception of the social impact of oral disorders on their well-being. Clarifying such an association is crucial since this may help us to better understand how OHRQoL serves as a critical factor for the risk of depressive symptoms in both genders. As gender differences in the associations between OHRQoL and emotional status in children and pre-adolescents have previously been observed [13], and the female gender has also been reported to be negatively correlated with OHRQoL in depression models [11], separate estimates for males and females were included in all the analyses and results.

In light of contemporary knowledge, a cross-sectional study was designed and aimed at investigating the gender-specific association between OHRQoL and depressive symptoms among college freshmen in China.

## Methods

### Study participants

In September 2017, a cross-sectional study was conducted on college freshmen from Shenyang Normal University in China. The research data were from the baseline investigation of an ongoing project of Fitness Improvement Tactics in Youths (FITYou), which aimed at clarifying the impact of socioeconomic, behavioral, physiological, and metabolic factors on the health-related quality of life and health status in college students.

A total of 4717 college students were invited to participate in this study, and 4323 subjects provided informed consent for their data to be analyzed (response rate: 91.6%), of which 218 did not participate in the health examination. Among these 4105 students, participants whose total scores of Self-rating Depression Scale (SDS) were unavailable (*n* = 383), and those whose total scores of OHIP-14 were missing (*n* = 58) were excluded from the analysis. Furthermore, we excluded participants with missing data in the variables of sex (*n* = 9), age (*n* = 33), body mass index (BMI) (*n* = 9), smoking status (*n* = 20), drinking status (*n* = 34), sleep duration (*n* = 59), frequency of breakfast (*n* = 21), parental education (*n* = 12), and number of siblings (*n* = 6). As a result, data from 3461 participants were used in this study (987 men and 2474 women). The process strictly abided by the spirit of the Helsinki Declaration [14], by protecting the rights and ensuring the safety of the participants. The study protocol was approved by the Ethics Committee of the College of Education, Zhejiang University.

### Assessment of OHRQoL

OHRQoL was evaluated by the 14-item Oral health impact profile (OHIP-14) [12], which was developed on the basis of OHIP-49 [15] by Slade et al. A total of 14 self-assessment items include five levels and correspond to the score (0 = no, 1 = very few, 2 = sometimes, 3 = often, 4 = very often). The total score ranges from 0 to 56, and the higher the score, the poorer the oral health status. To ensure adequate statistical power in each category, we categorized the subjects into three groups according to the total score of OHIP-14: Total score = 0, 1–6, and > 6, so that the responses in each category were generally similar.

### Assessment of depressive symptoms

Depressive symptoms were assessed by the Self-rating Depression Scale (SDS) [16], which is a self-rating scale reflecting the subjective feelings of depressed patients quite intuitively. The SDS contains 20 items, each of which is divided into a 4-level score to evaluate the main symptoms by frequency. The standard is: “1” indicates no or very little time; “2” means sometimes; “3” means most of the time; and “4” means most or all of the time. Of the 20 items, ten are stated in negative terms and the remaining ten in positive words, which are scored in reverse. The total scores of 20 items are added to get the initial score, then multiplied by 1.25 to arrive at the integer, which is the standard score. According to the Chinese version of the SDS, a standard score greater than 53 is identified as indicating depressive symptoms, while less than 53 identifies no depressive symptoms [17].

### Relevant covariables

For sociodemographic factors, the following variables were collected by a self-administered questionnaire. Age was calculated on the basis of birthdate and date of anthropometric examination. Ethnicity was estimated as Han or minority, based on the responses to a multiple-choice question. Further, sibling numbers were dichotomized into 0 or none with a response of “yes” or “no” to the question: “Are you the only child in your family?” The possible responses to education level of parents rating were: “Elementary school, junior high school, high school, junior college, undergraduate, master, or doctor” and were divided into two categories: ≥ high school or not, with the highest degree categories of both parents recorded as parental education level finally. Lifestyle-related information in health behaviors containing questions about smoking status (current, former, or never) and drinking status (current drinker, non-drinker) were obtained directly by multiple choices of this questionnaire. Frequency of breakfast was assessed by the following question: “In the last month, how often did you eat breakfast each week on average?” The eight alternative responses ranged from 0 to 7 times per week, which were dichotomized into more than five times per week or not. Sleep duration was assessed by the question: “In the last month, how many hours did you sleep every night?” with the possible answers categorized into four groups: < 6, 6–7, 7–8, > 8 h. The anthropometric parameter of BMI was calculated using the formula of weight in kilograms divided by height in square meters (kg/m^2^), which was measured in health examinations by medical practitioners. BMI was then categorized into three groups: < 18.5 (underweight), 18.5–24.9 (normal), and ≥ 25 kg/m^2^ (overweight), in accordance with classifications reported by the World Health Organization (WHO) [18].

### Statistical analysis

To describe gender-specific participants’ characteristics according to OHRQoL categories, an analysis of variance, and a chi-squared test were used for continuous and categorical variables. Percentages were presented for dichotomous variables, and arithmetic means (standard deviations [SD]) were presented for continuous variables, as appropriate.

To determine the association of gender-specific OHRQoL with depressive symptoms, logistic regression analyses were used to indicate odds ratios (ORs) and a 95% confidence interval (CI) for depressive symptoms in each category of OHRQoL, and then compared them to the categories with total scores of OHIP-14 equal to 0, which indicates better OHRQoL. Multivariable regression analyses included three statistical models adjusted for covariates. Model 1 consisted of age, ethnicity, number of siblings, and parental education level. Further to this, Model 2 added lifestyle-related variables including smoking status, drinking status, frequency of breakfast, and sleep duration. In addition to the covariates in Model 2, BMI was entered into Model 3. All tests were 2-tailed, and *P* < 0.05 was considered statistically significant. IBM SPSS Statistics 23.0 software for MAC (IBM Corp, Armonk, NY, USA) was used for statistical analysis in this study.

## Results

### Baseline characteristics of the participants

As is shown in Table 1, the characteristics of the subjects in the different categories of total score of OHIP-14 are summarized. A total of 3461 college students with ages ranging between 15 and 22 years (mean ± SD: 18.4 ± 1.3), participated in the study, consisting of 987 (28.5%) male students and 2474 (71.5%) female students. A high number of students (84.3%) were from the Chinese Han population. Overall, 72.3% of youths reported having fair or poor OHRQoL. One hundred and eighty-five male students and 532 female students had depressive symptoms, accounting for 20.7% of all study participants.

**Table 1:** Baseline characteristics of the participants in relation to OHRQoL

Variable ^a^	Total score of OHIP-14 (male, *n* = 987)			*P* value ^b^	Total score of OHIP-14 (female, *n* = 2474)			*P* value ^b^
	= 0	1–6	> 6		= 0	1–6	> 6	
Participants, no. (%)	299 (30.3)	379 (38.4)	309 (31.3)		659 (26.6)	973 (39.3)	842 (34.0)	
Age (years)	18.8 ± 1.7	18.6 ± 1.2	18.7 ± 1.4	0.050	18.3 ± 1.1	18.3 ± 1.2	18.3 ± 1.2	0.606
Han ethnicity, no. (%)	257 (86.0)	333 (87.9)	270 (87.4)	0.753	557 (84.5)	817 (84.0)	683 (81.1)	0.147
BMI (kg/m^2^), no. (%)								
< 18.5 (underweight)	43 (14.4)	54 (14.2)	52 (16.8)		132 (20.0)	183 (18.8)	191 (22.7)	
18.5–24.9 (normal)	173 (57.9)	221 (58.3)	200 (64.7)		438 (66.5)	651 (66.9)	550 (65.3)	
≥ 25 (overweight)	83 (27.8)	104 (27.4)	57 (18.4)	0.047	89 (13.5)	139 (14.3)	101 (12.0)	0.250
No sibling, no. (%)	215 (71.9)	246 (64.9)	187 (60.5)	0.012	400 (60.7)	552 (56.7)	432 (51.3)	0.001
Breakfast consumption ≥ 5 times a week, no. (%)	233 (77.9)	279 (73.6)	213 (68.9)	0.043	527 (80.0)	741 (76.2)	589 (70.0)	< 0.001
Smoking status, no. (%)								
Never	261 (87.3)	321 (84.7)	262 (84.8)		654 (99.2)	968 (99.5)	832 (98.8)	
Former	16 (5.4)	26 (6.9)	16 (5.2)		2 (0.3)	5 (0.5)	7 (0.8)	
Current	22 (7.4)	32 (8.4)	31 (10.0)	0.651	3 (0.5)	0 (0.0)	3 (0.4)	0.202
Current drinkers, no. (%)	82 (27.4)	128 (33.8)	125 (40.5)	0.003	31 (4.7)	61 (6.3)	62 (7.4)	0.106
Parental education level ≥ high school, no. (%)	131 (43.8)	148 (39.1)	93 (30.1)	0.002	239 (36.3)	337 (34.6)	229 (27.2)	< 0.001
Sleep duration (hours/day), no. (%)								
< 6	77 (25.8)	93 (24.5)	97 (31.4)		117 (17.8)	231 (23.7)	206 (24.5)	
6–7	101 (33.8)	149 (39.3)	106 (34.3)		241 (36.6)	361 (37.1)	319 (37.9)	
7–8	90 (30.1)	104 (27.4)	88 (28.5)		228 (34.6)	289 (29.7)	251 (29.8)	
> 8	31 (10.4)	33 (8.7)	18 (5.8)	0.155	73 (11.1)	92 (9.5)	66 (7.8)	0.010

*BMI* Body mass index, *SDS* Self-rating depression scale, *OHIP-14* Oral health impact profile-14

^a^ Percentages were presented for dichotomous variables, and arithmetic means (standard deviations [*SD*]) were presented for continuous variables

^b^ Analysis of variance or χ^2^ test

Overall, the associations between the characteristics of participants and OHRQoL were generally consistent in both genders. The participants who had no siblings (*P* < 0.05), whose frequency of eating breakfast was more than five times a week (*P* < 0.05), and who had parents with a higher level of education than a high school degree (*P* < 0.01), had significantly better OHRQoL, however, there were still some differences in both sexes. The percentage of participants with BMI more than 25 kg/m^2^ was lower in male students with poorer OHRQoL (*P* < 0.05), while males with no drinking habits had significantly better OHRQoL (*P* < 0.01). Shorter sleep duration (< 6 h) was associated with poorer OHRQoL, and the percentage of sleep duration of more than 8 h was lower in female students with poorer OHRQoL (*P* < 0.05). However, no significant differences were found in age, proportion of Han ethnicity, or smoking status in the different categories of OHRQoL.

### OHRQoL and depressive symptoms

**Fig. 1:**
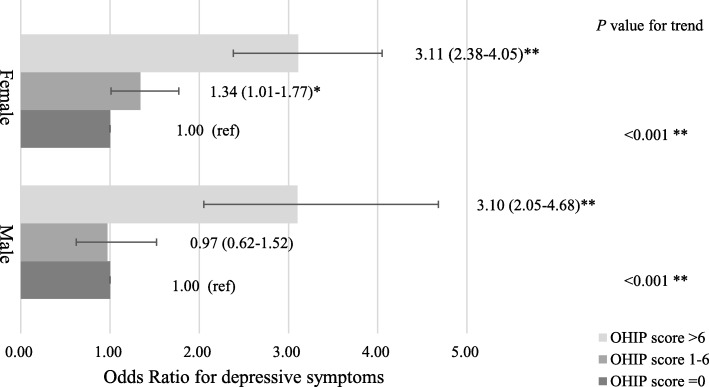
Non-adjusted odds ratio and 95% confidential interval for association between OHRQoL and depressive symptoms. Analysed by logistic regression analysis. Of the 3461 subjects, 717 had depressive symptoms. ^a^ The higher total scores of OHIP-14 indicate poorer OHRQoL. * *P* < 0.05,  ** *P* < 0.001

Figure 1 shows the association between OHRQoL and depressive symptoms in both genders before adjusting potential covariates. Compared to the category with a 0 score of OHIP-14 (good OHRQoL), the prevalence of depressive symptoms was higher in categories with a score of 1–6 (fair OHRQoL) in females for the univariate model (ORs [95% CI]: 1.34, 1.01–1.77, *P* = 0.041), although the discrepancy disappeared after adjusting all potential covariates shown in Table 2 (ORs [95% CI]: 1.28, 0.96–1.69, *P* = 0.092). The prevalence of depressive symptoms did not differ between these two categories in males (ORs [95% CI]: 0.94, 0.59–1.50, *P* = 0.798). While the prevalence of depressive symptoms was significantly higher in the category with a score over 6 (poor OHRQoL) (male: ORs [95% CI]: 3.07, 1.98–4.76, *P* < 0.001; female: ORs [95% CI]: 2.90, 2.21–3.81, *P* < 0.001). Similar results were observed in the multivariable analysis after adjusting all potential covariates. Therefore, it is necessary to conduct analytic researches on the association between OHRQoL and depressive symptoms.

**Table 2:** Logistic regression analysis for the association of OHRQoL with depressive symptoms

Sex	Total score of OHIP-14 ^a^	No. Depressive symptoms	Model 1^b^		Model 2^c^	Model 3^d^		
			ORs (95% CI)	*P* value	ORs (95% CI)	*P* value	ORs (95% CI)	*P* value
Male	0 (*n* = 299)	39	1.00 (ref)		1.00 (ref)		1.00 (ref)	
	1–6 (*n* = 379)	48	0.97 (0.61–1.53)	0.894	0.94 (0.59–1.50)	0.800	0.94 (0.59–1.50)	0.798
	> 6 (*n* = 309)	98	3.19 (2.10–4.86)	< 0.001	3.09 (2.00–4.77)	< 0.001	3.07 (1.98–4.76)	< 0.001
Female	0 (*n* = 659)	89	1.00 (ref)		1.00 (ref)		1.00 (ref)	
	1–6 (*n* = 973)	168	1.34 (1.01–1.77)	0.040	1.28 (0.96–1.69)	0.091	1.28 (0.96–1.69)	0.092
	> 6 (*n* = 842)	275	3.16 (2.41–4.12)	< 0.001	2.89 (2.20–3.80)	< 0.001	2.90 (2.21–3.81)	< 0.001

*OHRQoL* Oral health-related quality of life, *ORs* Odds ratios, *CI* Confidential interval

^a^ The higher scores indicate poorer oral health status

^b^ Adjusted for age (continuous), ethnicity (Han ethnicity, minority ethnicity), sibling number (0, ≥ 1 siblings), and parental education level (< high school, ≥ high school)

^c^ Same as Model 1 + smoking status (current, former, never), drinking status (current drinker, non-drinker), frequency of breakfast (< 5, ≥ 5 times per week), and sleep duration (< 6, 6–7, 7–8, > 8 h)

^d^ Same as Model 2 + body mass index (< 18.5, 18.5–24.9, ≥ 25 kg/m^2^)

## Discussion

This cross-sectional population-based study found that after adjusting for some sociodemographic and health-related behavior confounders, college students with poorer OHRQoL were more likely to have depressive symptoms. Furthermore, the current study is one of the few studies that has investigated the correlation between OHRQoL and depressive symptoms in youth.

The overall prevalence of depressive symptoms in the participants was 20.7%, with female students at 21.5% and male students 18.7%, respectively. These results are consistent with previous studies conducted at an American university [19] and among Chinese college students [20]. The high prevalence of depressive symptoms reflects the seriousness of psychiatric problems in college students. Although only some of the literature regarding association between OHRQoL and depressive symptoms in youths has been reported [10, 11], these previous findings are in agreement with our results, which illustrates that OHRQoL is inversely related to depressive symptoms. Alternatively, evidence has been reported that other mood disorders such as anxiety, negative affectivity, depression, and somatization [21] are related to OHRQoL.

The results of the analysis indicate that there is no relation between OHIP and depressive symptoms by sex (*P* for interaction = 0.685). Compared to the category with a 0 score of OHIP-14 (good OHRQoL), the prevalence of depressive symptoms was significantly higher in the category with a score over 6 (poor OHRQoL) in both genders for both the univariate model and in the multivariable analysis. Furthermore, the results show no significant difference between the prevalence of depressive symptoms in the categories with a score of 1–6 (fair OHRQoL) and a score of 0 after adjusting all potential covariates between the sexes. However, gender differences appeared when comparing the prevalence of depressive symptoms for the univariate model, which was higher in the categories with a score of 1–6 (fair OHRQoL) in females as compared to males. This may be explained on the basis of subsequent research that self-focus differs between the genders, which might moderate the effects of OHRQoL on depressive symptoms in a gender-specific manner. Self-focus refers to when an individual focuses his attention on his own emotions, thoughts, attitudes, behaviors and other parts that are related to his self-content, instead of focusing on work content and other people’s opinions, and external or self-irrelevant information [22]. A research conducted in college students found female students tended to focus more on themselves and their mood when depressed than male students [23]. Based on this evidence, separate estimates for both males and females were included in the analysis and results. Moreover, a logistic regression analysis was performed to establish the association of OHRQoL with depressive symptoms in all students in the fully adjusted model between the OHIP score of 0 and the OHIP score of 1–6 (*P* = 0.136), and between the OHIP score 0 and the OHIP score > 6 (*P* < 0.001), which were similar with gender-specific results.

These findings suggest that OHRQoL may have an influence on depressive symptoms through different pathways, which provides evidence to deepen understanding of the possible underlying mechanisms. Several potential mechanisms could explain the association of OHRQoL with depressive symptoms. Oral malodor has a mediated relationship between OHRQoL and depressive symptoms through the pathway of social psychiatry. A recent study reported that poorer OHRQoL was associated with oral malodor, dysgeusia, and burning sensations on the tongue [24]. Evaluated by the 14-item Oral health impact profile (OHIP-14) [12], the relationship between OHRQoL and halitosis may be due to painful aching, unsatisfactory diet, or inability to function. First, certain inflammatory cytokines are known to be involved in the initiation and persistence of pathologic pain [25], and oral infections with their imbalance of microorganisms can also be responsible for oral malodor [26]. Second, unsatisfactory diet, or having to interrupt meals because of problems with teeth, mouth, or dentures could lead to eating disorders, which has previously been confirmed as associated with gastrointestinal symptoms [27]. Gastrointestinal symptoms especially may still persist in psychologically distressed patients chronically [28], which may cause oral malodor [26]. Third, malfunctions such as taste disturbance, difficulty chewing or swallowing, or speech problems are all associated with salivary gland dysfunction [29], which can be responsible for oral malodor [30]. Oral malodor could further affect the interpersonal communication of millions of people psychosocially and lead to poor self-image, dwindling self-esteem and confidence, loneliness, and depression [31]. An alternative viewpoint by Vettore [32] was that decreased self-respect because of poor oral health was a source of increasing social isolation, which may lead to depression. Besides, poor oral health has an effect on speech problems and communication, which may adversely affect self-esteem and self-confidence [33]. The idea of “words as social tools” proposes that words could be a tool for communication with great social impact, thus struggling to pronounce words because of oral problems eliminates the tool function of word use and interpersonal relationships would suffer [34]. Together with the acknowledgment of an association between impaired social relationships and depressive symptoms [35], OHRQoL could influence the prevalence of depressive symptoms through lower self-esteem and life satisfaction due to barriers in interpersonal communication [36].

Furthermore, OHRQoL could also play a role in the development of depressive symptoms through neurobiological pathways facilitated by dysgeusia. As has already been mentioned, poor OHRQoL is associated with dysgeusia [24], which might be one of several factors leading to poor appetite and subsequent malnutrition [37]. Some indicated that depressive symptoms could be secondary to low spirits due to poor appetite and malnutrition, which is as a result of a reduction in the neuronal production of monoamines, especially serotonergic neurotransmission [38], and anhedonia [39]. A cross-sectional study supported this perspective by showing a strong association between taste dysfunction and depressive symptoms among American adults [40]. Another mediation may contribute to stress. According to the Surgeon General, poor OHRQoL can lead to chronic stress [41], which can cause depression [42]. The evidence supports that reduced neurosteroid allopregnanolone could result in hyperactivity of the hypothalamic-pituitary-adrenal (HPA) axis function, increased glucocorticoid levels, and reduced expression of brain-derived neurotrophic factor under chronic stress, and could further induce depression-like behaviors [43]. Other epidemiologic evidence of links between poor oral health and cognitive impairment, which predicts long-term depressive symptoms after stroke, was presented [44]. Alternatively, some results confirmed that microbiota could activate neural pathways and central nervous system (CNS) signaling systems through stress-induced change in the microbiota-gut-brain axis [45], which indicates the existence of an important association between microbiota and mental disorders, including anxiety and depression.

This research is limited by the fact that all participants were drawn from a convenient sample of Chinese youths from the Shengyang Normal university, which may undermine the ability to make generalizations from the sample to other populations with different ethnicities and ages. Further, the OHIP-14 is a short version of a self-rated instrument, and thus cannot reflect oral health conditions as comprehensively as a longer version. Moreover, in order to control for possible confounding factors, a considerable number of sociodemographic, anthropometric, and lifestyle behavioral parameters were taken into consideration. Nevertheless, it is undeniable that we cannot exclude the possibility that residual confounding, such as dietary habits and blood inflammatory biomarkers, still exists. Additionally, although both OHRQoL and depressive symptoms were assessed by standardized scales with high reliability and validity [12, 16], recall bias due to using self-reported questionnaires remains a possibility. Still, given the cross-sectional study design, it is difficult to make causal inference and reverse causation cannot be ruled out; thus, further exploration for association between OHRQoL and depressive symptoms is needed.

## Conclusions

In conclusion, this cross-sectional study has verified that OHRQoL is one of the critical factors for the risk of depressive symptoms in Chinese college freshmen. These findings suggest the importance of improving OHRQoL to prevent depressive symptoms and have helped raise awareness in, and concerns about, prevention strategies of depressive symptoms. A further longitudinal follow-up study is warranted to confirm the association between OHRQoL and depressive symptoms.

## Data Availability

The datasets used and/or analyzed during the current study are available from the corresponding author on reasonable request.

## Abbreviations

CI: Confidence interval
CNS: Central nervous system
FITYou: Fitness Improvement Tactics in Youths
OHIP-14: Oral health impact profile
OHRQoL: Oral health-related quality of life
ORs: Odds ratios
SD: Standard deviations
SDS: Self-rating Depression Scale
WHO: World Health Organization
